# Nitrogen Excretion, Ammonia, and Greenhouse Gases Emission in Italian Heavy Pigs: The Role of Feed in Environmental Impact Mitigation

**DOI:** 10.3390/ani16030520

**Published:** 2026-02-06

**Authors:** Raffaella Rossi, Eleonora Buoio, Edda Mainardi, Annamaria Costa

**Affiliations:** Department of Veterinary and Animal Sciences (DIVAS), University of Milan (UNIMI), Via dell’Università, 6, 26900 Lodi, LO, Italy; raffaella.rossi@unimi.it (R.R.); edda.mainardi@unimi.it (E.M.); annamaria.costa@unimi.it (A.C.)

**Keywords:** heavy pig, dietary interventions, nitrogen, gaseous pollutants, environmental footprint assessment

## Abstract

This review examined the environmental impact of fattening pigs, with particular focus on the Italian heavy fattening pig, affected by dietary interventions aimed at mitigating nitrogen excretion and gases emission into the atmosphere, maintaining product quality and complying with the regulation frameworks. In the Italian heavy pig supply chain, environmental regulations are often difficult to comply with due to the constraints imposed by PDO. The pig sector is increasingly committed to developing strategies that can effectively mitigate its environmental impacts. In intensive pig farming, emissions of ammonia (NH_3_) and greenhouse gases (GHGs) can occur in every production phase, from animal housing to manure treatment, management, storage, and up to in-field application. All these phases present key actions to limit emissions of NH_3_ and GHG, according to Best Available Techniques (BAT) for housing and Best Practices (BP) for manure treatments and management. Nitrogen excretion in manure is another important aspect to consider for its negative effects when applied in Vulnerable Zones with considerable additions of animal waste and synthetic fertilisers. Nutritional strategies can contribute a *priori* to mitigate nitrogen excretion, NH_3_ emissions, and overall GHG output in heavy pigs, particularly in Italian PDO ham systems. While these interventions effectively mitigate NH_3_ and influence manure-related emissions, their impact on total GHG varies depending on diet composition, fibre type, additive combination, and post-excretion treatment.

## Simple Summary

The focus of pig breeding in Italy is on producing heavy pigs, which are slaughtered at around 160 kg of live weight to produce Protected Designation of Origin (PDO) dry-cured products. The breeding of pigs is strictly regulated by the official guidelines to produce Parma and San Daniele dry-cured hams. In addition to the differences in nutrition, given the longer time spent on the farm, heavy pig production and PDO supply chains pose significant environmental challenges. Concerns have been raised about environmental issues, such as nitrogen emissions and greenhouse gas emissions. To address these issues, several dietary strategies have been proposed as an alternative to traditional ones. The focus of this review is on dietary interventions for reducing emissions during the fattening phase of Italian heavy pigs.

## 1. Introduction

Within the European pig farming sector, which mainly produces pigs for fresh meat, Italy stands out for the production of heavy pigs whose requirements differ from the standardised intensive systems used in the European Union. In 2024, Italy’s pig farming sector produced over 1.4 million tonnes of meat and accounted for 4.7% of the national agro-zootechnical economic value, highlighting its importance within the livestock industry [1].

This production strategy results from decades of refinement in genetics, nutrition strategies, and management, in accordance with official guidelines for Parma and San Daniele ham production [2]. Pigs are reared to an average slaughter weight of about 160 kg (±10%) at about 9 months of age, and the nutritional strategies influence carcass and thigh traits, their suitability for curing, and the physicochemical and sensory quality of dry-cured ham [3,4,5]. From a genetic point of view, the lines and hybrids have been selected by the Italian National Association of Pig Breeders to improve prime cut yield, skeletal strength, and optimal subcutaneous fat deposition while maintaining progress in growth performance, feed efficiency, prolificacy, and longevity [6]. The objective of Italian heavy pig breeding is to produce animals whose meat is suitable for processing into PDO products rather than to achieve rapid and higher growth rates for fresh meat consumption.

From a management perspective, the longer production cycle of heavy pigs requires careful control of dietary management, density, ventilation, microclimate, and animal health [7,8,9]. Nutritional strategies play a crucial role in pigs, supporting growth performance, feed efficiency, gut health, and immune system function [10,11,12]. Proper nutrition is crucial in heavy pig production using restricted feeding and medium-protein content diets, which vary according to the different growth stages [13]. Moreover, PDO specifications restrict raw materials during the growing and fattening phases to prevent soft fat [14].

The Italian heavy pig system poses a unique environmental challenge, as dietary management, slaughter weights and extended production cycles substantially impact climate and ecosystem quality. Given that nutritional solutions have also been proposed as a key strategy due to their potential to impact emission sources in heavy pig production [15], this review provides a comprehensive summary of studies examining the relationship between nutrition and feeding strategies in heavy pigs and their impact on mitigating the environmental footprint. This impact is examined in relation to dietary interventions aimed at mitigating nitrogen excretion and gas emissions, maintaining product quality and complying with regulatory frameworks.

## 2. Livestock Farming and Legislation Related to Mitigation Measures

Attention has been given to gaseous pollutants, such as ammonia and GHG, and nitrogen (N) and phosphorus (P) released into the environment by livestock farming and animal management, due to growing environmental concerns [16].

Early regulations for intensive livestock farming address local and regional issues to limit undesired processes with negative repercussions for the environment, such as eutrophication from N runoff and NH_3_ emissions. A key measure is the Directive 91/676/EEC (Nitrates Directive [17]), transposed in Italy, which aims to prevent and reduce nitrate pollution of surface- and groundwater from agricultural sources, particularly in vulnerable zones, present mostly in the Po Valley, through mandatory action programs.

The Gothenburg Convention (1999) established national emission ceilings through the NEC Directive (2001/81/EC), updated by Directive (EU) 2016/2284, which sets reduction targets for NH_3_, Nitrogen oxides, Sulfur Oxides, Non-Methane Volatile Organic Compounds, and fine particulate matter [18,19,20]. In Italy, it is implemented via Legislative Decree 152/2006, Decree 81/2018, and the National Air Pollution Control Programme (PNCIA).

Regulatory focus later expanded to greenhouse gas emissions, as IPCC reports highlighted livestock contributions to CH_4_ and N_2_O, supporting international agreements such as the Kyoto Protocol and the Paris Agreement [21,22]. More recently, EU initiatives like the European Green Deal and the Fit for 55 packages target a 55% GHG reduction by 2030 (vs. 1990 levels), fostering greater integration of climate objectives into agricultural and environmental policies despite no direct obligations for livestock farms.

Intensive pig farming is regulated under the IPPC Directive, later incorporated into the Industrial Emissions Directive, which requires large farms to apply Best Available Techniques (BATs) for intensive pig and poultry rearing [23,24,25]. These measures target NH_3_ reduction, improved nutrient efficiency, optimised manure management, and lower N losses to air, water, and soil, further reinforced by Directive (EU) 2024/1785 [26].

In particular, BATs highlight that nutritional management in pigs is a preventive measure aimed at enhancing the efficiency of dietary nitrogen retention in body tissues. In fact, it has been reported that in pigs slaughtered at 110 kg live weight (LW), only about 33% of ingested nitrogen is retained in body tissues, while the remaining fraction is excreted in manure. Consequently, various nutritional strategies have been proposed to mitigate nitrogen excretion, including multiphase feeding, the incorporation of feed additives to reduce nitrogen losses, the reduction in dietary crude protein, and the supplementation of synthetic amino acids to meet the animals’ nutritional requirements.

To achieve environmental mitigation, management practices, including nutritional strategies, must be applied through an integrated approach encompassing all aspects of the production system.

## 3. Focus on Pig Production and on the Modern Italian Heavy Fattener: The Nitrogen Balance and Its Excretion in Manure

Considering this scenario, the Italian PDO standards have adapted to the new challenges of emissions mitigation, introducing the updated Parma Ham regulations, allowing for greater flexibility in feed formulation—at least 50% of dry matter from local product and increasing the proportion of alternative protein sources, such as peas, from 5% to 25% of dry matter [27,28].

### 3.1. Diet, the Nitrogen Balance, and Excretion in Manure

In pigs, only a proportion of the N ingested from the diet is utilised for growth and metabolism. The excess is excreted in urine and faeces, with roughly two-thirds in urine and one-third in faeces [29]. Within urine, about 80% of N is present as urea, while the remainder consists of NH_3_ and other soluble nitrogen compounds [30]. In faeces, N that has not been absorbed by the intestines is primarily present in the form of proteins and complex organic compounds. The transformation of these molecules into ammonium ions (NH_4_^+^) is a relatively slow process requiring microbial enzymes and is facilitated by bacterial communities responsible for mineralising organic N [31]. Urea is more readily degradable than complex faecal compounds, being rapidly hydrolysed into NH_3_ and bicarbonate by urease [30,32]. Due to its production by various faecal microorganisms, including *Streptococcus* spp., *Actinomyces* spp., and *Lactobacillus* spp., urease is present in high concentrations in pig manure, significantly accelerating the process of ureolysis, particularly under optimal conditions of pH 6.7–7.0 and temperatures around 35 °C [30,31,32].

Nutrition plays a critical role in improving its efficiency in its utilisation, increasing faecal N excretion and ammonia emissions by 10% and urinary nitrogen by 3% while reducing dietary crude protein by 1% [33]. The optimal diet administration, within the so-called precision feeding technique, tailored to physiological and production needs, allows for reducing lysine intake by 25%, with a reduction in N excretion by 40% and feed costs by 8% over the fattening period (Pomar and Remus, 2019) [34].

In the past, pigs presented an efficiency in N utilisation of about 27–33% of ingested N, while the remaining 73–67% was excreted [31]. Similar trends have been observed across Europe, with pigs exhibiting N use efficiency ranging from 43 to 47% [32].

Specifically, today’s pigs have, on average, a higher feed intake and greater weight gain than those of 25 years ago, with a similar feed conversion index. This results in greater weights at slaughter than for pigs in the major PDO circuits for the production of hams, which must necessarily take place after 9 months of age, with average live weight up to 160 kg. Consequently, the N balance in these modern genetic types is different from that in the past, mainly due to a greater efficiency of N utilisation, taking to different environmental challenges and to different environmental impact assessments. As a result, the N balance of modern genetic types of pigs has a higher level of N retained (36.6% vs. 27.3%) [35]. Kasper et al. [36] reported 49% in the traditional diet and 47% in the CP-reduced diet, demonstrating a lower amount of excreted nitrogen compared to the nitrogen balance of the pigs of the past.

Furthermore, it should be noted that the length of the breeding period remains unchanged, and with this lower daily N excretion, there is the potential for a further improvement in N excretion per kilogram of meat produced.

Specifically, the N excretion and efficiency vary throughout the growing period. The N efficiency is highest during early growth (60–90 kg LW) and gradually decreases in later stages, with the lowest efficiency (around −6%, [36]) from 120 to 140 kg LW, corresponding to lower N retention and higher N intake [37].

### 3.2. Gaseous Pollutant Emissions in Pig Farming: The Role of Housing, Plants, Management/Treatment, and Spreading in the Field

So, although essential for nutrient cycling, excess soil N increases the risk of nutrient imbalance, soil acidification, and eutrophication. Nitrate not taken up by plants can leach into water bodies, causing pollution and contributing to N_2_O emissions, the most powerful GHG for global warming contribution [38].

The environmental impact of pig systems is the sum of many factors, with feed production identified as the main contributor (approximately 70%), followed by manure management (around 20%), direct emissions from animals (approximately 5%), energy use (approximately 3%), and other processes (approximately 2%) [39,40].

Although pig farming is responsible for relatively low levels of GHG emissions (about 9%) compared to other livestock farming, it is important to maintain efforts to make pig farming more sustainable. The main GHGs associated with pig production are CH_4_, carbon dioxide (CO_2_), and N_2_O, which are major contributors to the carbon footprint and global warming potential. One of the main environmental issues associated with pig farming is the excretion of nitrogen, which leads to NH_3_ emissions (about 14% from livestock and 8% of total human-caused NH_3_ emissions), soil acidification, eutrophication of sensitive ecosystems and odour problems, as well as issues relating to animal welfare [41]. Improving feed efficiency and reducing feed waste are key strategies for reducing overall emissions from pig production [42,43].

The environmental impact of pig production has been assessed in several studies, but these mainly refer to light pigs slaughtered at around 110–120 kg LW [44]. Analyses of conventional pig production evidence that the finishing stage (65 kg to slaughter weight) is the biggest contributor to the pig system’s environmental impact [45]. Studies have reported that pig fattening units have an environmental impact up to ten times greater than weaning units due to the longer breeding period, greater feed consumption, and less efficient use of nutrients compared to pigs at other stages and higher manure production [46,47,48]. Measures to mitigate emissions in the pig sector can usually be categorised into four distinct areas: animal-related, feed-related, building/plant/housing-related and manure-related. Improving feed efficiency is the primary key to mitigating NH_3_, CH_4_ and N_2_O emissions. This can be achieved by improving the general health of pigs and implementing genetic improvements to boost feed efficiency, nutrient adsorption, and growth [49]. It is also reported that it is essential to valorise pig slurry by coupling thermal, biological and phototrophic processes while simultaneously recovering energy and nutrients and mitigating greenhouse gas emissions [50].

Regarding housing, structures, and adopted plants, intensive pig farming is subject to regulations aimed at limiting their environmental impact by adopting BATs. BATs are defined in the IPPC as “*the most effective and advanced stage in the development of activities and their methods of operation, which indicate the practical suitability of particular techniques for providing, in principle, the basis for emission limit values designed to prevent and, where that is not practicable, generally to reduce emissions and the impact on the environment as a whole*”. The BATs are large-scale, sustainable methods from the economic point of view, created to guarantee the highest level of protection for the environment, mainly from NH_3_, which represents a critical gaseous pollutant on pig farms, produced when N compounds excreted in pig slurry are degraded via the hydrolysis of urea, by the action of urease [29,30].

In fresh pig manure, urea can be fully converted to NH_3_ within 96 h, causing a rapid increase in total ammoniacal nitrogen and a concomitant rise in pH, which enhances volatilisation [51]. Once formed, NH_4_^+^ dissociates into NH_3_, which volatilizes and contributes to emissions, with the rate influenced by pH, temperature, manure properties, and farm management [52].

**Figure 1:**
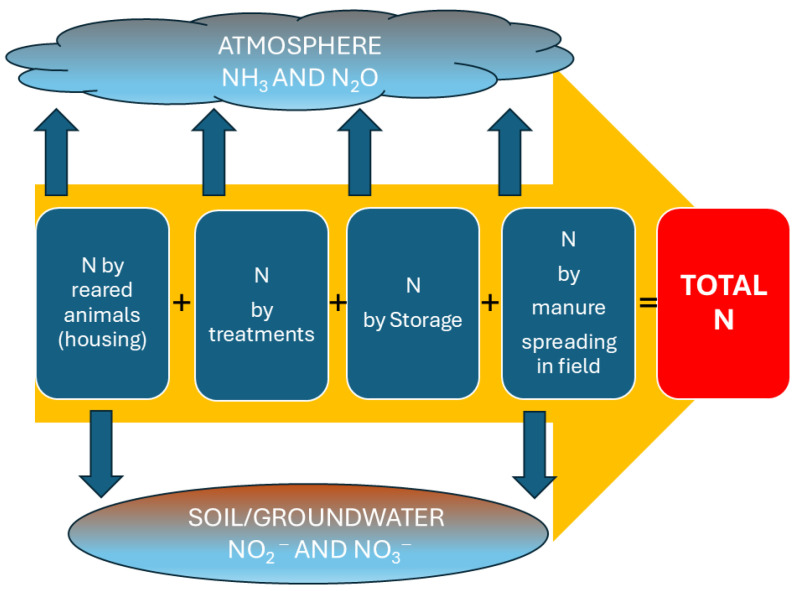
Pollutants generated in farms during the various phases of production and manure treatment-storage-spreading.

According to IPCC guidelines, Tier 1, the lowest level of accuracy for emission factor estimates, reports about 8–10% of excreted nitrogen volatilized as NH_3_ from housing and manure, while about 1% is lost as N_2_O during storage and field application [53,54]. The GHG emissions from stored manure increase with temperature from 18 °C to 28 °C by 74% for fresh manure and 66% for digestate [55]. Once in the soil, N becomes available for microbial processes within the N cycle. The NH_4_^+^ can be oxidised to nitrate (NO_3_^−^) through nitrification, and nitrate can then be reduced to nitrogen gases (N_2_, N_2_O) via denitrification [56]. In general, pollutant emissions in any farm occur at every stage of production, as shown in Figure 1, starting from the animal and the diet administered, up to the field.

There are mitigation methods for each phase of pollutant emissions. Nowadays, the key action in intensive livestock production is to strike a balance between maximising production and mitigating environmental and energy impact. Although emissions from the pig production stage are relatively small compared to total agricultural emissions, the intensive nature of pig farming and its concentration in specific geographical areas contribute to significant local emissions of N and NH_3_. This causes significant environmental damage to groundwater and surrounding areas. The leaching of nitrogen (nitrates) from the soil due to excessive application of synthetic slurry and fertilisers can lead to eutrophication of waters and ecosystems. In addition, some N excretion is converted into NH_3_, which causes soil and water acidification and negatively affects biodiversity in sensitive areas, reducing their ability to capture carbon and thus increasing total GHG emissions. NH_3_ is also a precursor to nitrogen oxide, a potent GHG, which further exacerbates emissions from livestock production [41].

Although pork generally has a lower per-unit GHG footprint than ruminant species, it is one of the most widely produced and consumed meats globally, resulting in a substantial overall environmental impact [57,58]. The main GHGs associated with pig production are CH_4_, CO_2_, and N_2_O, all contributing significantly to the carbon footprint and global warming potential of pork.

The CO_2_ emissions from pigs mainly originate from respiration and are part of the natural biological carbon cycle; therefore, they are generally not considered anthropogenic. The CO_2_ emissions from manure management, especially during composting, contribute to the farm’s carbon footprint. Most CO_2_-equivalent emissions arise from feed production (~70%) and on-farm energy use (~3%), which are typically accounted for in life cycle assessments [39,40]. Improving feed efficiency or reducing reliance on high-emission ingredients, such as soymeal, can indirectly reduce CO_2_-equivalent emissions. The CO_2_ from pig respiration (E-CO_2pig_) can be estimated according to body weight (LW) using the following equation, according to live weight (LW, in kg) [59].

E-CO_2pig_, in kg/day = 0.136 × LW^0.573^

Manure typically contributes 4–5% of total CO_2_ emissions, though site-specific conditions may increase this proportion [60].

The CH_4_ emissions in pigs originate from two main sources: enteric fermentation, occurring primarily in the cecum and large intestine, and anaerobic decomposition of manure during storage and handling. Enteric CH_4_ production in pigs is relatively low compared to ruminants, and is strongly influenced by diet composition, fibre content, and the efficiency of intestinal microbiota, which tends to increase with animal age [59].

At a mechanistic level, enteric CH_4_ production (E-CH_4_) in pigs can be estimated using feed-based models. For example, the INRA-AZN, 2004 model estimates CH_4_ emissions as a function of digestible residues (dRes) according to the following relationship [61].

E-CH_4_ = 0.0012 × dRes (g/day)

Such approaches explicitly link CH_4_ formation to diet digestibility and fermentable substrates and therefore go beyond the use of fixed emission factors. Empirical measurements indicate that enteric CH_4_ emissions for growing pigs range from approximately 2.5 to 4.1 g CH_4_/day [53,59]. These emissions correspond to 0.1–3.3% of gross energy intake, depending on diet composition, breed, and rearing system [62]. For national GHG inventories, Tier 1 is the standard approach for swine, reflecting the relatively small contribution of enteric methane to total emissions and the limited availability of country-specific feed data. Under Tier 1, emission factors are set at 1.5 kg CH_4_/head/year for high-productivity systems and 1.0 kg CH_4_/head/year for low-productivity systems [53]. Total methane emissions for a livestock category are calculated using the following equations [54]:

$$E_{T}=\sum_{\left(P\right)}EF_{\left(T,\;P\right)}\;\cdot \;\left(\frac{N_{\left(T,\;P\right)}}{10^{6}}\right)$$

where E_T_ is the CH_4_ emissions from enteric fermentation in category T (Gg CH_4_/year), N_T,P_ is the number of animals in productivity system P, and EF_T,P_ is the corresponding emission factor (CH_4_/head/year).

Overall enteric methane emissions (Gg CH_4_/year) are then obtained by summing across categories and productivity systems:

$$Total\;{CH}_{4\;enteric}=\sum_{i,P}E_{i,P}$$

where E_i,P_ represents the annual enteric methane emissions (Gg CH_4_/year) from the livestock category or subcategory “i” under production system “P”, calculated as the product of the number of animals and the corresponding emission factor.

In contrast, manure management represents the largest source of CH_4_ in pigs, with emissions averaging 32.9 g CH_4_/pig/day in European conditions and varying with storage type, duration, and environmental factors [53]. Although Tier 2 and Tier 3 methods can incorporate detailed feed intake data, energy conversion factors (Ym), and mechanistic fermentation models, these approaches are rarely applied to pigs due to the limited relevance of enteric CH_4_ and the scarcity of country-specific dietary data. Globally, pork production has a lower CH_4_ footprint per gram of protein (55 g CO_2_-eq/g protein) compared with ruminants such as buffalo (404 g CO_2_-eq/g protein) or beef (295 g CO_2_-eq/g protein) [57].

The N_2_O emissions from livestock manure originate primarily from microbial nitrification and denitrification processes, mediated by heterotrophic, facultative aerobic microorganisms during manure storage and treatment and following land distribution. The balance between these processes is strongly regulated by oxygen availability, redox potential, moisture content, and the availability of readily degradable carbon substrates. In conditions of limited oxygen and low labile carbon, the accumulation and release of N_2_O is favoured over complete reduction to N_2_ [53,54]. The N_2_O emissions are not directly measured but are estimated using emission factors (EFs) that are specific to manure management systems, accounting for both direct and indirect emissions resulting from nitrogen volatilisation (NH_3_ and NO_x_) and subsequent atmospheric deposition, as well as N leaching and runoff. This cascade approach reflects the progressive transformation and redistribution of excreted nitrogen across the agricultural system [54]. Accordingly, total N_2_O emissions associated with N excretion from a given animal type (T) are calculated as the sum of emissions arising from manure management, land application of manure, and excreta deposition on pasture, range and paddock systems, as expressed by

$$N_{2}O_{\left(T\right)}=N_{2}O_{mm\left(T\right)}+N_{2}O_{AM\left(T\right)}+N_{2}O_{PRP\left(T\right)}$$

where N_2_O_mm(T)_ represents direct and indirect N_2_O emissions from manure management systems, N_2_O_AM(T)_ arises from the application of manure to managed soils, and N_2_O_PRP(T)_ represents emissions from excreta deposited during grazing. Emissions from manure management are estimated by combining system-specific emission factors for direct N_2_O formation with fractions of nitrogen lost via volatilisation and leaching pathways, according to

$$N_{2}O_{mm\left(T\right)}=\left(\sum_{S}^{.}F_{mm\left(T,S\right)}\cdot \left[EF_{3\left(S\right)}+{\left(Frac_{gasMS}\right)}_{{\{}_{\left(T,S\right)}}\right\}\cdot EF_{4}+{\left(Frac_{leachMS}\right)}_{{\{}_{\left(T,\;S\right)}}\right\}\cdot EF_{5}\cdot \frac{44}{28}$$

where *F_mm(T,S)_* is the annual nitrogen flow associated with animal type *T* in manure management system *S*, *EF_3_* is the direct N_2_O emission factor for manure management, *Frac_GasMS_* and *Frac_LeachMS_* are the fractions of nitrogen volatilized or leached, respectively, and *EF_4_* and *EF_5_* are the corresponding indirect emission factors. All emissions are initially expressed as N_2_O–N and subsequently converted to N_2_O using the molecular weight ratio (44/28).

## 4. Dietary Interventions

Research aimed at improving the sustainability of the pork sector has long focused on alternative feed ingredients, diet formulation and precision feeding strategies [63,64]. Among the available mitigation levers, nutritional interventions represent one of the most powerful tools, as they directly influence the two primary sources of emissions: feed-related impacts and manure excretion. In Table 1, an overview of feeding strategies evaluated for the mitigation of nitrogen excretion and gaseous emissions in livestock systems is shown.

### 4.1. Reduction in Dietary Crude Protein in Heavy Pigs

In this context, the reduction in dietary crude protein (CP) combined with the supplementation of amino acids (AA) has been widely promoted for several decades as an effective strategy to decrease N excretion and related NH_3_ emissions while simultaneously reducing the reliance on imported soybean meal—a key driver of land-use changes and deforestation [15,75]. Soybean meal is currently the predominant protein source in pig diets, owing to its high digestibility, favourable amino acid profile, and consistent global availability. However, the European livestock sector’s reliance on soybean meal is associated with several critical issues, including price volatility, environmental degradation and deforestation. In addition, feed production represents the main contributor to greenhouse gas emissions in pig farming systems. Consequently, reducing the environmental footprint associated with soybean meal has become a strategic priority. In this context, increasing scientific interest is being directed toward alternative protein sources capable of providing comparable nutritional value while significantly lowering environmental impact [76].

Several studies have demonstrated that adopting low-CP diets, when properly balanced with essential amino acids, allows the maintenance of growth performance, carcass traits, and product quality in the heavy pig fattening phase [15,28,79]. Lowering the CP content of pig diets reduces the breakdown of excess AA, thereby decreasing N excretion and the environmental impact of N emissions. As CP levels decrease, so does nitrogen excretion, with a linear relationship that can be quantified at around 8% per point of CP reduction, and at the same time, NH_3_ emissions drop by 10% per point of CP reduction [34,80].

This evolution in swine nutrition reflects a broader trend towards precision feeding, driven by a deeper understanding of amino acid metabolism and requirements. Advances in amino acid science have enabled more accurate and effective diet formulations, aiming to balance growth promotion with environmental sustainability [81].

The effects of low-protein diets on N balance in fattening phases of heavy pigs have been explored, considering the impact of dietary CP level on growth performance, carcass characteristics and meat quality. It has been observed that low-protein diets without AA supplementation increased carcass fatness compared with the control diet [82]. However, when indispensable AA levels were maintained similar to those in control diets, carcass fatness was not affected by low-CP diets [83,84]. A recent review suggests that there is a minimum crude protein level (10.3–12.5%) for finishing pigs, after which pigs may have compromised growth performance, even if their diet is balanced for all essential AA [85]. Moreover, in the fattening phase, there is a limit of 6.5% for the total Lys/CP, and below this value, protein intake may not be sufficient [86].

It is reported that carcass characteristics and the weights and yields of the main lean cuts in heavy pigs were not affected by fattening diets containing 120 or 99 g CP/kg feed, with total lysine maintained at 6.5 g/kg feed [87]. Lowering dietary CP by 15% compared to the control diet had no significant effect on carcass traits or ham weight in heavy pigs [79]. Another study reported that a diet with low CP content (−3% less than the control diet) and supplemented with lysine (6.5 g/kg feed) did not affect growth performance and carcass quality in heavy pigs, but decreased N excretion by 24.5% compared to controls [88]. Also, another study reported that, in the fattening phase, lowering dietary CP (from 13% to 9%), while ensuring the indispensable AA level through the administration of synthetic AA, did not affect ham quality parameters [89]. A recent study reported that in the fattening phase, a low-protein diet (10% vs. 10.8% control diet) with the addition of essential AA reduces N excretion (−28% than control) and enhances N efficiency without affecting production or meat quality parameters [87]. Overall, these data highlight the potential to reduce the environmental impact of N excretion during the fattening phase of heavy pigs without significantly affecting productive parameters or pork quality.

### 4.2. Increasing Dietary Fibre in Heavy Pigs

Increasing dietary fibre in heavy pigs can modulate gut fermentation, shift N excretion from urine to faeces, and reduce NH_3_ emissions from manure. In fact, in finishing diets, fibre sources, such as wheat bran and beet pulp, affect energy and N balance and may influence nutrient digestibility [90].

Fibre-rich diets improve gut health by promoting beneficial microbial populations and enhancing fermentation, which may support nutrient absorption and reduce N losses [69]. Furthermore, an increase in fermentation caused by feeding high-fibre diets results in higher levels of short-chain fatty acids in faeces. This, in turn, leads to a decrease in the pH value of slurries, which in turn reduces NH_3_ losses [91]. The reduction in NH_3_ emissions is particularly beneficial when beet pulp and soybean hulls are included [90,91]. The inclusion of wheat bran and oat hulls in the finishing diet is less important for reducing NH_3_ emissions because they are less fermented in the gut [90]. A diet was formulated to include maximum levels of wheat bran (200 g/kg) and beet pulp (40 g/kg) in substitution for barley meal [79]. This was done in accordance with the Consortia guidelines to study the effects of a high-fibre diet on nitrogen excretion. Animals fed the high-fibre diet excreted more N in faeces than pigs fed the control diet, with no difference in urinary N excretion. Moreover, no differences in growth performance and carcass yield were observed [79]. Previous studies conducted with heavy pigs have shown that the inclusion of a high level of fibrous ingredients (240 g wheat bran or 300 g sugar beet pulp/kg of diet) negatively affects carcass yield [92,93].

It is crucial to consider potential compromises when applying nutritional mitigation strategies in pig production. In fattening light pigs, high-fibre diets containing sugar beet pulp have been shown to significantly reduce ammonia (NH_3_) emissions from housing and manure (23.2 vs. 45.0 g) while simultaneously increasing enteric methane (CH_4_) production (37.9 vs. 27.2 g). Moreover, an impairment of growth performance and carcass traits was also observed [94]. These contrasting effects highlight the need for a holistic approach to diet formulation that balances multiple environmental objectives with animal performance and carcass traits.

### 4.3. Use of Dietary Additives in Heavy Pigs

The addition of enzymes (e.g., phytase, xylanase) improves nutrient digestibility and reduces the excretion of phosphorus and undigested fractions, with indirect benefits for waste management. It should be noted that no single additive or dietary intervention can guarantee a substantial reduction in emissions. Conversely, a combination of CP reduction, AA supplementation, and the use of enzymes and functional additives, acting synergistically, appears to be the most promising strategy for improving the environmental sustainability of intensive pig farming [95]. One of the first nutritional solutions to be studied was the use of exogenous enzymes. The use of enzymes such as phytase and carbohydrases (e.g., xylanase and β-glucanase) has been a well-established strategy for improving nutrient digestibility and increasing the efficiency of P and fibre fractions in the diet, limiting the amount of undigested nutrients in waste [96]. However, improved digestive efficiency and reduced fermentable fractions in wastewater can indirectly contribute to lower NH_3_ and GHG production during manure storage and management.

In addition to additives acting at the digestive level, other nutritional strategies target the physicochemical characteristics of excreta, thereby influencing emissions mainly during manure handling and storage rather than at the enteric level. These include bentonite, a natural clay belonging to the phyllosilicates, widely used in animal feed for its adsorbent properties. In the pig farming sector, the use of bentonite is authorised as a technological additive and is part of the nutritional strategies aimed at improving food safety and digestive matrix stability [97]. Inclusion of 10 g bentonite/kg in the heavy pig did improve N utilisation [79]. Bentonite does not directly affect nutrient digestibility or enteric fermentation. However, it reduces the availability of N that is easily volatilised in manure. Therefore, including it in pig diets can be useful in reducing the potential for NH_3_ emissions during slurry storage and application to land.

As reported in Table 1, several dietary interventions have been shown to affect nitrogen excretion and ammonia emissions, even when tested in pigs slaughtered at lighter weights than Italian heavy pigs. Prebiotic supplementation, including seaweed polysaccharides and oligosaccharides, can beneficially alter gut microbiota and fermentation in pigs, enhancing nutrient digestibility and potentially reducing N emissions [98]. Moreover, dietary inulin supplementation in growing–finishing pigs improves growth performance and carcass yield, likely through modulation of intestinal fermentation and microbial activity [99]. Further research is needed to determine the impact of dietary supplements on nitrogen N and GHG emissions during the fattening phase of heavy pigs.

### 4.4. Alternative Ingredients

Alternative ingredients are essential tools to reduce N excretion, NH_3_ emissions, and overall GHG output in heavy pig production [14,100]. In particular, considering the PDO’s regulatory limits imposed on dietary ingredients and levels of inclusion, alternative protein sources such as sunflower meal and peas have been promoted as partial replacements for soybean meal. Although limited data are available for heavy pigs, two experimental trials have provided some useful information. In the first study, the replacement of soybean meal with sunflower meal and peas during the pig fattening phase (CP 54% in Control vs. 11.30% in experimental groups) did not impair growth performance and only slightly affected the faecal microbial profile, with no significant impact on faecal NH_3_ concentration [100]. In the second experimental trial, pigs were fed a protein pea-based diet as a replacement for soybean meal, and barley and sorghum as a replacement for maize [14]. The diets were iso-energetic and iso-proteic and were balanced for the ratio of lysine D/digestible energy. Even though no differences were observed in productive performance, nitrogen excretion or meat quality parameters, these results provide a basis for testing new dietary formulations during the fattening phase of heavy pigs.

Given the need to reduce the environmental impact of Italian pig farming, these findings are important and warrant further investigation.

Integrating multiple dietary strategies, such as reducing crude protein, supplementing synthetic amino acids to meet nutrient requirements, and including fibre sources, additives, or alternative ingredients permitted under PDO regulations, provides a practical approach to optimise both production efficiency and environmental sustainability in heavy pig farming. These strategies can reduce N excretion and NH_3_ emissions, improving gut health and immune function, without affecting growth performance and carcass traits. Overall, a holistic dietary approach is essential to balance productive, health, and environmental objectives in heavy pig production systems.

## 5. The Environmental Footprint of the Italian Heavy Pig: A Scenario

### 5.1. The Nitrogen Footprint in Relation to Diet and Nitrogen Efficiency Utilisation

**Figure 2:**
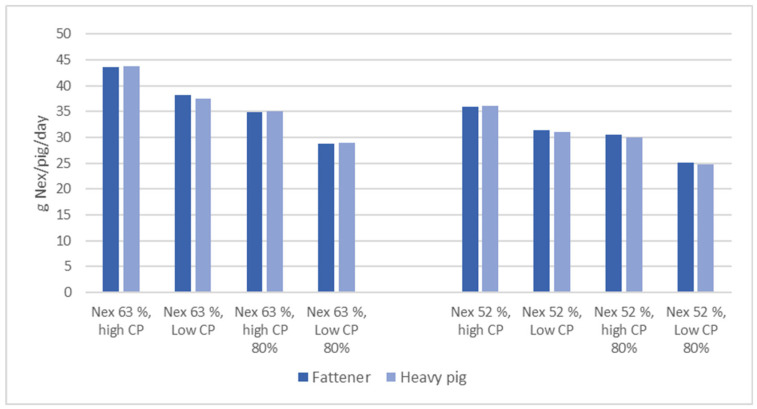
Scenario of nitrogen excretion in POD, according to N excretion and CP variation. (Nex: % nitrogen excretion on N intake).

To produce a scenario of N footprint of the Italian heavy pig, Figure 2 reports the amount of N produced, expressed in kg/ton of LW/year, calculated on the input of CP/N in the diet.

In this scenario, a traditional diet for the pigs was considered according to PDO nutritional guidelines (high CP 16%, low CP 14% for fatteners and high CP 14%, low CP 12 for heavy pig diets), with different animal N excretion efficiency, 63% and 52% (the modern selected pig), according to the literature [101], to provide a nitrogen balance for the different categories.

The diets were also submitted to a further 20% CP reduction, according to Bee et al. [102], for all the production phases; the results are shown in Figure 2.

It is evident that the genetic selection of pigs able to retain N more efficiently leads to a lower N impact on the environment, with similar results obtained by reducing CP at 80%. The reduction in CP during the last two fattening phases did not produce particular benefits in improving N footprint for traditional pigs with Nex of 63%, but some positive effects can be reached, coupling animals with Nex of 52% to a CP-restricted diet in the last two fattening phases.

In this scenario, the heavy pigs of PDO show a very similar amount of N excreted per day, in comparison to fatteners. The sum of the impact of heavy pigs vs. fatteners, as N excretion, during all the production cycle from the weaners’ phase, is higher around 40%, as was expected for the almost doubled LW in comparison with lighter fatteners.

So, for the longer time spent on the farm, heavy pig production and PDO supply chains pose further environmental challenges, given that pork is the most widely produced and consumed meat globally [57,58].

### 5.2. The Environmental Impact of PDO: Ammonia and GHG Emissions

In this last section, the ammonia and GHG emissions are simulated according to the fattener and heavy pig characteristics.

The data were calculated using the BAT tool Plus software [103]. The software allows us to calculate gas emissions of the farm according to the nutrition level, type of manure storage, treatment and spreading on the field, starting from excreted nitrogen, which is subtracted from the losses of ammonia (expressed as ammoniacal nitrogen, N-NH_3_) from the shelter. The remaining nitrogen arrives at the treatment (if present), where it is subject to ammonia nitrogen losses from the treatment phase; the remaining nitrogen goes to storage, where it is subject to ammonia nitrogen losses from the storage phase, and the remaining nitrogen arrives at the agronomic distribution, where it is subject to ammonia nitrogen losses from this phase. The sum of the losses of ammoniacal nitrogen (converted to ammonia by multiplying by the ratio of molecular weights 17/14) from the four phases constitutes the overall loss of the farm.

Regarding the values shown in Figure 1, in general, nitrogen losses occur from the animal house (13%) under gaseous ammonia form, from storage tanks (15%), from treatments, if done, and from manure spreading, volatilized as ammonia (10–25%) and for leaching in soil (20–35%).

Data related to nitrous oxide are taken from the IPCC *Manual Good Practice Guidance and Uncertainty Management in National Greenhouse Gas Inventories*, according to Tier 2, CH_4_ emissions from enteric fermentation come from the 2006 IPCC Guidelines for National Greenhouse Gas Inventories, Chapter 10, *Emissions from Livestock and Manure Management* [53,54].

To compare fatteners to heavy pigs’ direct environmental impact, considering also the nutritionally reducing CP strategy, data are calculated keeping into account reference or “non-BAT”, housing, storage and spreading conditions, and data are reported in Table 2.

Table 2 reported the value of ammonia emission, related to pigs reared in a traditional box, on a deep pit, that resulted in a 13% CP reduction in the diet, both for fatteners and heavy pigs.

CO_2_ eq balance did not differ, considering the enteric fermentation of animals, according to housing and management.

## 6. Conclusions

This manuscript highlights the importance of housing, targeting feeding strategies, and manure management during the finishing period to achieve effective mitigation of N, NH_3_, and GHG. In the Italian heavy pig supply chain, environmental regulations are often difficult to comply with due to the constraints imposed by PDO regulations. The pig sector is increasingly committed to developing strategies that can effectively mitigate its environmental impacts. Therefore, light pig management cannot simply be applied to Italian pig breeding, which focuses on heavy pigs. Differences in final weight, cycle duration, carcass composition, growth requirements, and the technological constraints imposed by PDO regulations result in specific nutritional needs.

In conclusion, the Italian pig sector can significantly reduce its environmental footprint through targeted nutritional strategies. Tailoring diets to each growth stage, optimising protein–energy balance, and incorporating functional ingredients or additives not only maintains growth performance and carcass quality but also lowers N excretion by around 20% and can reduce ammonia and GHG emissions. These approaches demonstrate that targeted nutritional strategies are a practical and effective tool for achieving both productive efficiency and environmental sustainability in heavy pig production.

## Figures and Tables

**Table 1:** Overview of feeding strategies evaluated for the mitigation of nitrogen excretion and gaseous emissions in livestock systems. The table summarises, for each strategy, the main technical aim, the corresponding IPCC category mapping, and the reported effects on nitrogen excretion, volatile solids excretion, and emissions of NH_3_, CH_4_, and N_2_O.

Feeding Strategy	Main Technical Aim	IPCC Category Mapping	Effect on NH_3_/N_ex_	Effect on CH_4_/VS_ex_	Effect on N_2_O	Ref.
**Lower CP + AA**	Match AA needs with less protein	↓N_ex_ (Tier 2)	↓NH_3_ 8–11% per % unit CP; 22–57% in trials	Small direct effect; some lower CH_4_ per kg of weight gained	Lower N_ex_ → lower N_2_O; weak/no direct change	[65,66,67]
**Fibre-rich diets**	Limit over-intake; gut N fixation	↓N_ex_; ↑faecal C	↓N_ex_ if protein optimised	↑VS_ex_; CH_4_ may increase	Lower N_ex_ per kg → lower N_2_O	[68,69]
**Prebiotics,** **Probiotics,** **enzymes**	Modify gut microbiota	Feed additives altering EFs	↓NH_3_	Some reduce CH_4_; product-specific	N_2_O rarely measured	[70,71]
**Shift N urine to** **faeces**	Reduce urea N in urine	N_ex_ similar; pH/TAN change	↓NH_3_ large (70% potential)	More fermentable C: ↑CH_4_	Total N_ex_ similar → Tier 2 N_2_O (same)	[69,70]
**Acidifying diets**	Lower urine/manure pH	↓NH_3_ EFs housing/storage	Some ↓NH_3_; evidence mixed	Little effect	Lower NH_3_ volatilisation→ less indirect N_2_O	[69,72]
**Zeolite**	Slurry chemistry	Feed additive	↓NH_3_ 25%	↓in-house CO_2_-eq 36%; storage ↑CH_4_ tendency	N_2_O unchanged	[71,73]
**Alternative** **local proteins**	Replace soybeans meal	Feed production emissions; N_ex_/VS_ex_	NH_3_ similar to low-CP. some ↓N_ex_	↓LU and, when avoiding deforestation/LUC, ↓GWP	Manure-chain N_2_O follows N_ex_	[74,75]
**Phase feeding**	Better match overgrowth phases	↓N_ex_ via better N supply	↓N_ex_ 15–30%; ↓NH_3_	Little direct effect	Lower N_ex_ → lower inventory N_2_O	[69]
**Precision feeding**	Daily real-time adjustment	↓N_ex_ (Tier 2)	↓N_ex_ 30–40%; ↑N-use efficiency	↓Feed use/kg; ↓VS_ex_ likely	Lower N_ex_ → lower inventory N_2_O	[76,77,78]

N_ex_ = N excreted, VS_ex_ = volatile solids excreted, CP = crude protein, AA = amino acids, TAN = total ammonia nitrogen, VS = volatile solids, EF = emission factors, LU = land use, LUC = land use change, GWP = Global Warming Potential, ↓ = reduction, ↑ = increase.

**Table 2:** Mean values of ammonia (NH_3_), methane (CH_4_), nitrous oxide (N_2_O) and carbon dioxide (CO_2_) (kg/pig/year) emitted by fatteners and the Italian heavy pig, depending on enteric fermentation.

	NH_3_	CH_4_ Enteric	CH_4_ Total	N_2_O	CO_2_ Enteric	CO_2_ eq Total
Fattener, Traditional diet	6.24	1.50	10.84	0	37.50	33.95
Heavy pig, Traditional diet	8.02	1.50	13.04	0	37.50	42.14
Fattener, Reduced CP 80%	5.39	1.5	10.48	0	37.50	32.76
Heavy pig, Reduced CP 80%	6.93	1.5	13.04	0	37.50	40.94

## Data Availability

Data is contained within the article.

## Abbreviations

The following abbreviations are used in this manuscript:

PDO	Protected Designation of Origin
GHG	Greenhouse Gas
BAT	Best Available Techniques
BP	Best Practices
IPPC	Integrated Pollution Prevention and Control
LW	Live weight
N_ex_	Nitrogen excreted
VS_ex_	Volatile solids excreted
CP	Crude protein
AA	Amino acids
TAN	Total ammonia nitrogen
VS	Volatile solids
EFs	Emission factors
LU	Land use
LUC	Land use change
GWP	Global Warming Potential
